# Risk of breast cancer in young women in relation to body size and weight gain in adolescence and early adulthood

**DOI:** 10.1038/sj.bjc.6690667

**Published:** 1999-09

**Authors:** R J Coates, R J Uhler, H I Hall, N Potischman, L A Brinton, R Ballard-Barbash, M D Gammon, D R Brogan, J R Daling, K E Malone, J B Schoenberg, C A Swanson

**Affiliations:** Division of Cancer Prevention and Control, Centers for Disease Control and Prevention, CDC, K-55, Atlanta, 30341-3717, USA; Division of Cancer Epidemiology and Genetics and; Division of Cancer Control and Population Sciences, National Cancer Institute, Bethesda, 20892, USA; Division of Epidemiology, Columbia School of Public Health, New York, 10032, USA; Department of Biostatistics, Emory University, Atlanta, 30322, USA; Division of Public Health Sciences, Fred Hutchinson Cancer Research Center, Seattle, 98104, USA; Cancer Epidemiology Program, New Jersey Department of Health and Senior Services, Trenton, 08652, USA

**Keywords:** breast neoplasms, weight gain, histological grade, neoplasm staging, adolescence, premenopause

## Abstract

Findings have been inconsistent on effects of adolescent body size and adult weight gain on risk of breast cancer in young women. These relations were examined in a population-based case control study of 1590 women less than 45 years of age newly diagnosed with breast cancer during 1990–1992 in three areas of the US and an age-matched control group of 1390 women. Height and weight were measured at interview and participants asked to recall information about earlier body size. Logistic regression was used to estimate the relative risk of breast cancer adjusted for other risk factors. Women who were either much heavier or lighter than average in adolescence or at age 20 were at reduced risk. Weight gain after age 20 resulted in reduced risk, but the effect was confined to early-stage and, more specifically, lower grade breast cancer. Neither the risk reduction nor the variation by breast cancer stage or grade was explained by the method of cancer detection or by prior mammography history. These findings suggest that relations between breast cancer risk in young women and body weight at different ages is complex and that the risk reduction with adult weight gain is confined to less aggressive cancers. © 1999 Cancer Research Campaign


British Journal of Cancer (1999) **81**(1), 167–174
© 1999 Cancer Research Campaign
Article no. bjoc.1999.0667

### Risk of breast cancer in young women in relation to body size and weight gain in adolescence and early adulthood

**RJ Coates[1], RJ Uhler[1], HI Hall[1], N Potischman[2], LA Brinton[2], R Ballard-Barbash[3], MD Gammon[4], DR Brogan[5], JR Daling[6], KE Malone[6], JB Schoenberg[7] and CA Swanson[2]**

[1]Division of Cancer Prevention and Control, Centers for Disease Control and Prevention, CDC, K-55, 4770 Buford Highway NE, Atlanta, GA 30341-3717, USA; [2]Division of Cancer Epidemiology and Genetics and [3]Division of Cancer Control and Population Sciences, National Cancer Institute, Bethesda, MD 20892, USA; [4]Division of Epidemiology, Columbia School of Public Health, New York, NY 10032, USA; [5]Department of Biostatistics, Emory University, Atlanta, GA 30322, USA; [6]Division of Public Health Sciences, Fred Hutchinson Cancer Research Center, Seattle, WA 98104, USA; [7]Cancer Epidemiology Program, New Jersey Department of Health and Senior Services, Trenton, NJ 08652, USA

**Summary** Findings have been inconsistent on effects of adolescent body size and adult weight gain on risk of breast cancer in young women. These relations were examined in a population-based case control study of 1590 women less than 45 years of age newly diagnosed with breast cancer during 1990–1992 in three areas of the US and an age-matched control group of 1390 women. Height and weight were measured at interview and participants asked to recall information about earlier body size. Logistic regression was used to estimate the relative risk of breast cancer adjusted for other risk factors. Women who were either much heavier or lighter than average in adolescence or at age 20 were at reduced risk. Weight gain after age 20 resulted in reduced risk, but the effect was confined to early-stage and, more specifically, lower grade breast cancer. Neither the risk reduction nor the variation by breast cancer stage or grade was explained by the method of cancer detection or by prior mammography history. These findings suggest that relations between breast cancer risk in young women and body weight at different ages is complex and that the risk reduction with adult weight gain is confined to less aggressive cancers.

**Keywords:** breast neoplasms; weight gain; histological grade; neoplasm staging; adolescence; premenopause

Adiposity or gaining weight increases risk of breast cancer in older, postmenopausal women but may reduce risk in premenopausal or young women (Ballard-Barbash, 1994). A recent meta-analysis of 23 studies of current body mass index (BMI) and risk of premenopausal breast cancer concluded that, although there was substantial heterogeneity in the results of these studies, BMI was inversely related to risk (Ursin et al, 1995). Research on breast cancer in young women is inconsistent in demonstrating whether risk is reduced with greater adiposity in early adulthood (ages 18–25) (London et al, 1989; Lund et al, 1990) or with subsequent weight gain (Taioli et al, 1995; Mannisto et al, 1996). There has been little research on effects of adolescent body size (Brinton and Swanson, 1992; Ursin et al, 1994).

In a case control study of breast cancer in women under the age of 45, we found that thin women had a greater risk of breast cancer than did heavy women (Swanson et al, 1996). The inverse relation was confined to early-stage breast cancer, but it did not vary by method of breast cancer detection, indicating that it was not explained by earlier detection among thinner women. The purpose of this current study was to examine risk of breast cancer in these young women in relation to adiposity in early adulthood and to subsequent weight gain. In addition, we examined risk in relation to perceived relative height and weight during adolescence and to changes in these perceived relative heights and weights.

*Received 14 October 1998*
*Revised 25 February 1999*
*Accepted 2 March 1999*

*Correspondence to:* RJ Coates

### MATERIALS AND METHODS

This case control study was conducted in three geographically defined locations in the USA, the metropolitan areas of Atlanta, Georgia and Seattle, Washington, and five counties in central New Jersey (Brinton et al, 1995). The protocol was approved by human subjects review boards at each collaborating institution. All women aged 20–44 years newly diagnosed with a first primary in situ or invasive breast cancer in these areas during 1 May 1990 to 31 December 1992, were rapidly identified through frequent review of hospital records. Of 1940 eligible cases identified, we obtained interviews from 1668 (86.0%). Reasons for non-interview included subject refusal (6.7%), physician refusal (5.8%), death or illness (0.8%) or other reasons (0.7%). Eighty per cent of the cases were interviewed within 6 months of diagnosis.

Control subjects were ascertained through 13 waves of random digit telephone dialing. Sampling probabilities were varied depending upon the age distribution of women in the areas and the projected distributions of cases. A short telephone screener to identify potentially eligible controls was completed at 90.5% of 16 254 telephone numbers assessed as residential, and a random sample stratified by 5-year age groups was obtained. Of 1989 controls selected, 82 were subsequently found ineligible, and interviews were completed on 1500 (78.7%). The overall response rate (screening times interview) was 71.2%.

Structured in-person interviews collected detailed information on breast cancer risk factors. To aid recall, a calendar method was used to record major life events. Cases were also asked how the breast problems leading to diagnosis were discovered and what treatments they had received. Information on risk factors was truncated at a common reference date, the patients' diagnosis dates and controls' screening dates.

Information on body size and weight gain was collected by anthropometric measurement and by interview. Standing height was measured using a stadiometer and weight by a portable digital scale (Seca Corp., Columbia, MD, USA). Participants were asked about their perceived relative heights and weights at interview and at ages 9–10, 12–13 and 15–16 years: 'Compared with other [girls/women] your age, would you say you were a) much shorter, b) somewhat shorter, c) about the same, d) somewhat taller, or e) much taller?' and 'Compared with other [girls/women] your age would you say you were a) much thinner, b) somewhat thinner, c) about the same, d) somewhat heavier, or e) much heavier?' The interview asked about weight in pounds at age 20, and at the heaviest and lightest weights subsequent to age 20. Women were asked where they tended to gain weight and how many times they had lost and then regained 15 or more pounds. The interview also asked how much weight was gained during each pregnancy. This study's findings of no relation between weight gain during pregnancy and breast cancer risk have been published (Troisi et al, 1998).

We created a number of body size and weight gain variables. BMI (weight (kg)/height (m)$^2$) was classified into approximate quintiles, and decile categorizations were created for a more detailed examination of dose–response relations. Total adult weight change variables were created by subtracting the weight at age 20 from highest, lowest and interview weights, and categories were created to provide a range of levels while retaining sufficient numbers for relatively stable risk estimates. To standardize the adult weight change for height, we calculated change in BMI. Because younger women may have gained weight at a rate similar to older women but had fewer years to gain, and because rate of weight change may have a different effect than total change, average yearly weight and BMI change variables were computed. For changes in perceived relative height and weight in adolescence, participants were categorized as having increased or decreased their relative height or weight in comparison with other girls if they changed categories from an earlier age period to a later one.

Tumour stage, oestrogen receptor status and histological grade (Percy et al, 1990) were obtained from the Surveillance, Epidemiology and End Results (SEER) program (Ries et al, 1997) records in Seattle and Atlanta. Stage and oestrogen receptor status were obtained from subjects' medical records in New Jersey. Stage was defined as in situ if the neoplasm was non-infiltrating, localized if the invasive neoplasm was confined entirely to the breast, regional if the tumour had extended directly beyond the breast into surrounding tissues or into regional lymph nodes, or distant if it had spread to sites remote from the primary tumour by direct extension or by discontinuous metastases.

For this analysis, we excluded 21 cases who were interviewed but who did not have a telephone at the time they were diagnosed, and 53 cases and 105 controls for whom we were unable to obtain either a weight or, less commonly, a height measure. Seven of these excluded cases and 46 excluded controls had not been measured because they were pregnant or less than 7 months postpartum.

Multivariate logistic regression (Breslow and Day, 1980; SAS, 1992) was used to estimate the relative risks of breast cancer associated with body size and weight change variables and their 95% confidence intervals adjusted for potential confounders. All models included study area and age (continuous). In addition, we adjusted for race (white, black, other), level of education (≤ high school, vocational/technical, some college, college graduate, post-graduate), family history of breast cancer (none, grandmother or half-sister, mother or sister), age at menarche (age ≤ 11, 12, 13, ≥ 14 years), previous breast biopsy (no, yes), number of term births and age at first term birth (no births, one birth at ≤ age 24, two births at ≤ age 24, three births at ≤ age 24, one birth at > age 24, two births at > age 24, three births at > age 24, and ≥ four births), years of oral contraceptive use (≤ 6 months, 7–59 months, 60–119 months and ≥ 120 months), recent alcohol consumption (none, < 1 per day, 1–2 per day, ≥ 2 drinks per day), height (quintiles) and number of mammograms obtained during the 5-year period prior to 1 year before reference date (0, 1, 2, 3+). In addition, we evaluated recency of oral contraceptive use, adolescent diet, smoking, menstrual status and whether or not menstrual cycles ever became regular as potential confounders. In this study, physical activity (Gammon et al, 1998) and adult macronutrient and calorie intakes (Potischman et al, 1997) had no effect on risk. Indicator variables were created for each category or level of each exposure variable and potential confounder. Trends in relations between categorical variables and risk were obtained by ordering the categories and entering an ordinal variable.

We examined possible variation in effect estimates among subgroups and used the likelihood ratio test statistic to determine statistical significance. Of particular interest was whether effects might vary by age at diagnosis, menopausal status, race, BMI at age 20, or age at menarche. In addition, to determine if the risks differed by breast cancer stage, method of cancer detection, or chemotherapy treatment, we conducted separate analyses for each case subgroup.

### RESULTS

Risk of breast cancer was inversely associated with BMI at interview (Table 1). Trends were observed whether BMI was categorized into quintiles or deciles, with risk reduced by approximately 2% for each unit increase in BMI. There was no linear trend in the relation between BMI at age 20 and risk, but risk appeared to be reduced among women whose BMIs were either greater or less than those of women in the middle quantiles. BMIs at the maximum and minimum weights attained between age 20 and reference date were unrelated to risk (data not shown).

Weight gain between age 20 and interview was inversely related to risk, with a trend toward a greater reduction with greater gain (Table 2). This was true whether weight gain was measured in kilograms or BMI units and whether women were categorized by total or average annual weight gain. Compared with women whose weights were relatively stable, those who gained more than 1.5 kg per year had approximately 35% less risk. Risk was reduced by approximately 2% for each unit increase in BMI after age 20 (data not shown). No effects were found for change in weight from age 20 to maximum or minimum weights, losing and then regaining 15 pounds or more, or location of weight gain (data not shown).

Relations between breast cancer risk and weight gain were not changed when BMI at age 20 was added to the regression models, but adjustment for current BMI reduced the associations (data not

© 1999 Cancer Research Campaign

**Table 1** Risk of breast cancer in relation to adult BMI and weight among women aged 20–44

| Characteristic | Cases No. % | Controls No. % | RR[a] | 95% CI[a] |
|---|---|---|---|---|
| BMI[b] at interview | | | | |
| 15.0–21.5 | 405 (25.5) | 278 (20.0) | 1.0 | |
| 21.6–23.6 | 303 (19.1) | 279 (20.1) | 0.76 | (0.60–0.96) |
| 23.7–25.8 | 301 (18.9) | 279 (20.1) | 0.74 | (0.58–0.93) |
| 25.9–30.3 | 309 (19.4) | 277 (19.9) | 0.79 | (0.62–0.99) |
| 30.4–54.9 | 272 (17.1) | 277 (19.9) | 0.69 | (0.54–0.88) |
| Trend RR across quintiles[c] | 1590(100) | 1390 (100) | 0.93 | (0.88–0.98) |
| Trend RR per BMI unit[d] | 1590 (100) | 1390 (100) | 0.98 | (0.97–1.00) |
| BMI at age 20 | | | | |
| 13.4–18.5 | 301 (19.0) | 278 (20.1) | 0.79 | (0.63–1.00) |
| 18.6–19.7 | 318 (20.0) | 276 (19.9) | 0.84 | (0.67–1.06) |
| 19.8–21.0 | 378 (23.8) | 278 (20.1) | 1.0 | |
| 21.1–22.7 | 315 (19.9) | 277 (20.0) | 0.85 | (0.68–1.07) |
| 22.8–44.2 | 275 (17.3) | 276 (19.9) | 0.75 | (0.59–0.95) |
| Trend RR by quintile[e] | 1587 (100) | 1385 (100) | 1.00 | (0.98–1.02) |
| Trend RR by BMI difference[f] | 1587 (100) | 1385 (100) | 0.94 | (0.91–0.98) |

RR = relative risk; CI = confidence interval. [a]Adjusted for age, study site, race, family history of breast cancer, previous breast biopsy, education, number of live births, age at first live birth, age at menarche, oral contraceptive use, history of mammograms, alcohol consumption and height. [b]Body mass index: [weight (kg)]/[height (m)]$^2$. [c]Risk associated with increase from a lower to a higher quintile category. [d]Risk associated with one unit increase in BMI. [e]Risk associated with a difference of one quintile, either higher or lower, from the middle (reference) quintile. [f]Risk associated with a one unit BMI increase or decrease from the median BMI of 20.3 in controls.

**Table 2** Risk of breast cancer in relation to change in weight and in body mass index[d] from age 20 to interview, among women age 20–44

| | Cases No. (%) | Controls No. (%) | RR[a] | (95% CI)[a] |
|---|---|---|---|---|
| Total weight change (kg) | | | | |
| Lost ≥ 3 | 84 (5.3) | 73 (5.3) | 0.79 | (0.53–1.17) |
| Gained/lost ± 2 | 204 (12.9) | 142 (10.3) | 1.0[b] | |
| Gained 3–5 | 202 (12.7) | 150 (10.8) | 0.93 | (0.68–1.27) |
| Gained 6–10 | 315 (19.9) | 287 (20.7) | 0.74 | (0.56–0.98) |
| Gained 11–20 | 425 (26.8) | 392 (28.3) | 0.76 | (0.59–1.00) |
| Gained ≥ 21 | 375 (22.5) | 341 (24.6) | 0.72 | (0.54–0.95) |
| Trend RR by category[c] | 1503 | 1312 | 0.93 | (0.87–0.98) |
| Trend RR per kg[c] | 1503 | 1312 | 0.99 | (0.99–1.00) |
| Annual weight change (kg) | | | | |
| Lost ≥ 0.3 | 52 (3.3) | 54 (3.9) | 0.64 | (0.42–0.98) |
| Gained/lost ± 0.2 | 378 (23.8) | 262 (18.9) | 1.0[b] | |
| Gained 0.3–0.5 | 376 (23.7) | 325 (23.5) | 0.80 | (0.64–1.00) |
| Gained 0.6–0.1 | 402 (25.3) | 372 (26.9) | 0.77 | (0.62–0.96) |
| Gained 1.1–1.5 | 215 (13.6) | 188 (13.6) | 0.82 | (0.63–1.06) |
| Gained ≥ 1.6 | 164 (10.3) | 184 (13.3) | 0.65 | (0.49–0.86) |
| Trend RR by category | 1535 | 1331 | 0.92 | (0.87–0.98) |
| Trend RR per kg | 1535 | 1331 | 0.89 | (0.79–0.99) |

RR = relative risk; CI = confidence interval. [a]Adjusted for age, study site, race, family history of breast cancer, previous breast biopsy, education, number of live births, age at first live birth, age at menarche, oral contraceptive use, history of mammograms, alcohol consumption and height. [b]Referent. [c]In the first row, risk is estimated for a change from a lower to a higher quintile category. In the second row, risk is estimated for a unit increase in BMI or weight (kg). Women in the weight loss category are excluded from the trend analysis.[d]Body mass index: [weight (kg.)]/[height (m)]$^2$.

shown). Adjusted for current BMI, the odds ratios for annual increases in BMI of 0.1, 0.2, 0.3–0.4 and ≥ 0.5 per year were increased from 0.91, 0.75, 0.79 and 0.68, to 0.95, 0.83, 0.87 and 0.74 respectively. Similarly, adjustment for annual increase in BMI increased the odds ratios for current BMI quintiles 2–5 from 0.76, 0.74, 0.79 and 0.69 respectively (Table 1) to 0.78, 0.79, 0.87 and 0.83 respectively.

Among women who recalled being much shorter than other girls when they were ages 12–13, risk of breast cancer was about 30% less (RR = 0.68) than it was among those who recalled being about

*© 1999 Cancer Research Campaign* 

**Table 3** Risk of breast cancer in relation to perceived relative height and weight during adolescence, among women age 20–44

| Perceived body size | Relative height | | | | | | Relative weight | | | | | |
|---|---|---|---|---|---|---|---|---|---|---|---|---|
| | Cases | | Controls | | | | Cases | | Controls | | | |
| | No. | (%) | No. | (%) | RRα | (95% CI)[a] | No. | (%) | No. | (%) | RRα | (95% CI) |
| **Age 9–10** | | | | | | | | | | | | |
| Much shorter/thinner | 103 | (6.5) | 121 | (8.7) | 0.78 | (0.58–1.05) | 301 | (18.9) | 283 | (20.4) | 0.91 | (0.73–1.12) |
| Somewhat shorter/thinner | 254 | (16.0) | 230 | (16.6) | 0.98 | (0.79–1.21) | 336 | (21.2) | 297 | (21.4) | 0.96 | (0.79–1.18) |
| About the same | 714 | (44.9) | 626 | (45.0) | 1.0 | | 627 | (39.5) | 523 | (37.6) | 1.0 | |
| Somewhat taller/heavier | 329 | (20.7) | 269 | (19.4) | 1.03 | (0.84–1.25) | 276 | (17.4) | 235 | (16.9) | 0.96 | (0.77–1.19) |
| Much taller/heavier | 189 | (11.9) | 144 | (10.4) | 1.10 | (0.85–1.43) | 49 | (3.1) | 52 | (3.7) | 0.70 | (0.46–1.08) |
| **Age 12–13** | | | | | | | | | | | | |
| Much shorter/thinner | 82 | (5.2) | 105 | (7.6) | 0.68 | (0.49–0.94) | 243 | (15.3) | 228 | (16.4) | 0.88 | (0.70–1.10) |
| Somewhat shorter/thinner | 270 | (17.0) | 262 | (18.9) | 0.92 | (0.74–1.13) | 324 | (20.4) | 291 | (20.9) | 0.91 | (0.74–1.11) |
| About the same | 749 | (47.1) | 649 | (46.7) | 1.0 | | 688 | (43.3) | 565 | (40.7) | 1.0 | |
| Somewhat taller/heavier | 341 | (21.5) | 245 | (17.6) | 1.17 | (0.96–1.43) | 290 | (18.3) | 252 | (18.1) | 0.90 | (0.73–1.11) |
| Much taller/heavier | 147 | (9.3) | 129 | (9.3) | 0.93 | (0.71–1.22) | 44 | (2.8) | 54 | (3.9) | 0.63 | (0.41–0.96) |
| **Age 15–16** | | | | | | | | | | | | |
| Much shorter/thinner | 78 | (4.9) | 94 | (6.8) | 0.73 | (0.53–1.01) | 189 | (11.9) | 204 | (14.7) | 0.73 | (0.58–0.93) |
| Somewhat shorter/thinner | 328 | (20.6) | 292 | (21.0) | 0.98 | (0.81–1.19) | 344 | (21.6) | 288 | (20.7) | 0.95 | (0.78–1.16) |
| About the same | 824 | (51.8) | 712 | (51.2) | 1.0 | | 779 | (49.0) | 631 | (45.4) | 1.0 | |
| Somewhat taller/heavier | 279 | (17.5) | 215 | (15.5) | 1.10 | (0.89–1.36) | 250 | (15.7) | 227 | (16.3) | 0.85 | (0.68–1.05) |
| Much taller/heavier | 81 | (5.1) | 77 | (5.5) | 0.92 | (0.66–1.30) | 28 | (1.8) | 40 | (2.9) | 0.53 | (0.32–0.88) |

RR = relative risk; CI = confidence interval. [a]Adjusted for age, study site, race, family history of breast cancer, previous breast biopsy, education, number of live births, age at first live birth, age at menarche, oral contraceptive use, history of mammograms and alcohol consumption. Perceived relative height is adjusted for perceived relative weight and vice versa.

**Table 4** Risk of breast cancer in relation to annual weight change age 20 to interview, by tumour grade, among women in Atlanta and Seattle, age 20–44

| Annual weight change from age 20 to interview (kg) | Cases[a] | | | Cases | | |
|---|---|---|---|---|---|---|
| | No. (%) | RR[b] Low-grade[e] | (95% CI)[b] | No. (%) | RR[b] High-grade[e] | (95% CI)[b] |
| Lost ≥ 0.3 | 5 (2.0) | 0.32 | (0.11–0.88) | 19 (4.3) | 1.05 | (0.55–2.02) |
| Gained/lost ± 0.2 | 68 (26.7) | 1.0[c] | | 79 (20.0) | 1.0 | |
| Gained 0.3–0.5 | 59 (23.1) | 0.65 | (0.42–1.00) | 95 (21.6) | 0.98 | (0.68–1.42) |
| Gained 0.6–0.1 | 67 (26.3) | 0.67 | (0.44–1.00) | 113 (25.7) | 0.92 | (0.64–1.31) |
| Gained 1.1–1.5 | 39 (15.3) | 0.94 | (0.57–1.56) | 66 (15.0) | 1.14 | (0.74–1.74) |
| Gained ≥ 1.6 | 17 (6.7) | 0.42 | (0.23–0.79) | 68 (15.5) | 1.09 | (0.71–1.68) |
| Total | 255 (100) | | | 440 (100) | | |
| Trend RR by category[d] | 250 | 0.89 | (0.78–1.01) | 421 | 1.04 | (0.94–1.14) |
| Trend RR per kg[d] | 250 | 0.81 | (0.63–1.03) | 421 | 1.09 | (0.91–1.28) |

[a]Histological data were not available for New Jersey cases. The distribution of weight change in the control group was lost ≥0.3 (35); no change (175); and gained 0.3–0.5 (218), 0.6–1.0 (274), 1.1–1.5 (128), and ≥1.6 (120). RR=relative risk; CI=confidence interval. [b]Adjusted for age, study site, race, family history of breast cancer, previous breast biopsy, education, number of live births, age at first live birth, age at menarche, oral contraceptive use, history of mammograms, alcohol consumption and height. [c]Referent. [d]In the first row, risk is estimated for a change from a lower to a higher category. In the second row, risk is estimated for an increase in 1 kg weight. Women in the weight loss category are excluded from the trend analysis. [e]Low-grade tumours are moderately and well differentiated; high-grade tumours are poorly differentiated or undifferentiated.

the same height as other girls their age (Table 3). However, there was no trend of increased risk with increased height. Women who recalled being much heavier were also at reduced risk, almost 50% less (RR = 0.53) for women who were much heavier at ages 15–16. Also, women who were much thinner than other girls at ages 15–16 were at reduced risk, although the effect was half that of being much heavier. There were no trends across the relative weight categories.

Changes in perceived relative weight were not associated with risk of breast cancer, and neither were changes in perceived relative height, with one exception. Among women who recalled becoming relatively taller compared with other girls at ages 15–16 than they were at ages 12–13, the risk of breast cancer was 0.72 times (95% CI 0.54–0.97) the risk among women who recalled being the same relative height at both ages (data not shown).

Adjustment for the breast cancer risk factors included in all models only modestly reduced the odds ratios for the body size measures we examined. This was true despite the fact that, for example, early menarche was associated with heavier weight at age 20, greater subsequent weight gain and heavier weight at interview. Similarly, the number of full-term births was positively associated with weight gain and BMI at interview, but not BMI at age 20. Although women who were either heavier or lighter at age 20, who subsequently gained more weight, or were heavier at interview were more likely to have never developed regular menstrual cycles, adjustment for menstrual regularity had no effect on the odds ratios.

Relations between breast cancer risk and the adolescent and adult BMI and weight gain variables did not vary by methods of breast cancer detection or treatment or by category of the

 © 1999 Cancer Research Campaign

**Table 5** Risk of breast cancer in relation to annual weight change age 20 to interview, by stage and by tumour grade, among women in Atlanta and Seattle, age 20–44

| Annual weight change from age 20 to interview (kg) | Cases[a] No. (%) | RR[b] Low-grade[e] | (95% CI)[b] | Cases No. (%) | RR[b] High-grade[e] | (95% CI)[b] |
|---|---|---|---|---|---|---|
| **In-situ and local stage** | | | | | | |
| Lost ≥ 0.3 | 2 (1.3) | 0.20 | (0.04–0.89) | 6 (2.9) | 0.71 | (0.27–1.87) |
| Gained/lost ± 0.2 | 44 (27.5) | 1.0[c] | | 41 (19.6) | 1.0 | |
| Gained 0.3–0.5 | 44 (27.5) | 0.80 | (0.49–1.31) | 47 (22.5) | 0.94 | (0.59–1.52) |
| Gained 0.6–1.0 | 35 (21.9) | 0.54 | (0.32–0.90) | 58 (27.8) | 0.93 | (0.59–1.48) |
| Gained 1.1–1.5 | 22 (13.8) | 0.81 | (0.44–1.50) | 29 (13.9) | 1.05 | (0.59–1.86) |
| Gained ≥ 1.6 | 13 (8.1) | 0.54 | (0.27–1.10) | 28 (13.4) | 0.92 | (0.52–1.66) |
| Total | 160 (100) | | | 209 (100) | | |
| Trend RR by category[d] | 158 | 0.87 | (0.74–1.01) | 203 | 0.99 | (0.87–1.32) |
| Trend RR per kg[d] | 158 | 0.84 | (0.63–1.13) | 203 | 1.01 | (0.80–1.27) |
| **Regional/distant stage** | | | | | | |
| Lost ≥ 0.3 | 3 (3.3) | 0.57 | (0.14–2.28) | 12 (5.4) | 1.31 | (0.59–2.90) |
| Gained/lost ± 0.2 | 24 (26.1) | 1.0‡ | | 37 (16.7) | 1.0 | |
| Gained 0.3–0.5 | 14 (15.2) | 0.42 | (0.20–0.88) | 46 (20.7) | 1.02 | (0.63–1.67) |
| Gained 0.6–0.1 | 31 (33.7) | 0.90 | (0.47–1.70) | 52 (23.4) | 0.90 | (0.55–1.46) |
| Gained 1.1–1.5 | 16 (17.4) | 1.10 | (0.51–2.37) | 37 (16.7) | 1.28 | (0.74–2.22) |
| Gained ≥ 1.6 | 4 (4.4) | 0.20 | (0.06–0.66) | 38 (17.1) | 1.26 | (0.72–2.19) |
| Total | 92 (100) | | | 222 (100) | | |
| Trend RR by category | 89 | 0.90 | (0.73–1.10) | 210 | 1.08 | (0.95–1.23) |
| Trend RR per kg | 89 | 0.69 | (0.46–1.05) | 210 | 1.16 | (0.94–1.43) |

[a]Histological data were not available for New Jersey cases. The distribution of weight change in the control group was lost ≥ 0.3 (35); no change (175); and gained 0.3–0.5 (218), 0.6–1.0 (274), 1.1–1.5 (128), and ≥1.6 (120). R = relative risk; CI = confidence interval. [b]Adjusted for age, study site, race, family history of breast cancer, previous breast biopsy, education, number of live births, age at first live birth, age at menarche, oral contraceptive use, history of mammograms, alcohol consumption and height. [c]Referent. [d]In the first row, risk is estimated for a change from a lower to a higher category. In the second row, risk is estimated for an increase in 1 kg weight. Women in the weight loss category are excluded from the trend analysis. [e]Low-grade tumours are moderately and well-differentiated; high-grade tumours are poorly differentiated or undifferentiated.

adjustment variables, but risk reduction with weight gain did vary by cancer stage (data not shown). Risk of in situ and of local stage breast cancer, but not regional/distant stage cancer, was reduced with increasing weight gain. As was the case for BMI at interview (Swanson et al, 1997), the variation in effect by stage was not dependent upon differences in method of breast cancer detection (by which we stratified) or by mammography history (which we included as an adjustment variable). Risks related to BMI at age 20 and to the adolescent exposures did not vary by stage.

Because weight gain was associated with early-stage cancer independently of the method of detection and of mammography screening history, it seemed plausible that the effect of weight gain might vary by tumour characteristics indicative of the rate of breast cancer progression. Lower grade cancers, those that are well- or moderately differentiated, have lower cell proliferation rates and slower progression than are higher grade cancers, those that are poorly differentiated or undifferentiated (Tabar et al, 1996). Similarly, oestrogen receptor-positive tumours may progress more slowly than oestrogen receptor-negative tumours (Habel and Stanford, 1993).

We found that the effects of weight gain after age 20 did not vary by oestrogen receptor status (data not shown), but they did vary by tumour grade (Table 4). Risk of lower grade breast cancer was reduced among women with the greatest weight gain, and there appeared to be a trend of reduced risk with greater gain. However, the protective effect of weight gain was not evident for higher grade tumours. Among women with ungraded cancers, 39% of which were in situ and 40% of which were local, there was an inverse relation between risk and weight gain (data not shown).

To determine whether the effect of weight gain was more influenced by grade or stage, we stratified cases by both and found that the relation appeared to be more dependent on grade than on stage (Table 5). There was no evidence of an inverse relationship between weight gain and risk of high-grade cancer whether it was in situ/local stage or regional/distant stage. In contrast, with in-situ/local stage cancer, there was a trend of reduction in risk of low-grade cancer with weight gain. For regional/distant stage, the number of low-grade cancers was small, but both the risk estimate for women who gained the most weight and the trend relative risks suggested a reduction in risk of low-grade cancer with weight gain.

### DISCUSSION

The results of this study of breast cancer in young women add to the evidence that women who gain weight after age 20 are at reduced risk and raise the possibility that women who were either much heavier or much lighter than average at age 20 are at reduced risk. While it is generally accepted that current BMI is inversely related to risk of breast cancer in young women (Ballard-Barbash, 1994; Ursin et al, 1995), it is unclear whether BMI in early adulthood (ages 18–25), subsequent weight gain, or both, contribute to this inverse association. None of the published studies reported that both women who were heavier and those who were lighter than average in early adulthood were at reduced risk. Some reported an inverse relation between risk and early adult BMI (Paffenbarger et al, 1980; Willett et al, 1985; London et al, 1989; Brinton and Swanson, 1992; Ursin et al, 1994; Huang et al, 1997; Trentham-Dietz et al, 1997), and others reported no relation (Choi et al, 1978; Pryor et al, 1989; Lund et al, 1990; Chu et al, 1991; Radimer et al, 1993; Ursin et al, 1994; Ziegler et al, 1996). Similarly, while some reported that weight gain resulted in reduced risk (Lubin et al, 1985; Le Marchand et al, 1988; London et al, 1989; Brinton and Swanson, 1992; Taioli et al, 1995; Franceschi et al, 1996), others did not (Paffenbarger et al, 1980; Lund et al, 1990; Chu et al, 1991; Radimer et al, 1993; Mannisto et al, 1996; Ziegler et al, 1996; Huang et al, 1997; Trentham-Dietz et al, 1997).

Both methodological differences among studies and error in assessment of early adult body weight may contribute to the inconsistency in this literature. Differences in study design, populations and analysis are associated with differences in findings on relations between current body size and breast cancer risk in young women (Pathak and Whittemore, 1992; Ursin et al, 1995). A detailed methodologic analysis of the literature on effects of early adult BMI and weight gain is beyond the scope of this discussion, but it is apparent from a brief review that neither cohort nor case control studies are consistent in their findings. For example, effects of weight gain have varied over time within the same cohort (London et al, 1989; Huang et al, 1997). Small study samples and differences in how BMI and weight gain were categorized and how trends were assessed may have contributed to some of the differences in findings. Also, although research suggests that weights at age 18 recalled by middle-aged adults correlate well with recorded weights (Casey et al, 1991; Huang et al, 1997), random error in recall is likely to contribute to the inconsistency. Since current BMI is a function of early adult weight and subsequent gain, error in recall of the early weight may affect whether, in addition to current BMI, either early adult BMI, subsequent weight gain, or both, are inversely related to risk.

Our finding that weight gain and current BMI were inversely related to risk of early- but not late-stage breast cancer is in agreement with a more consistent literature. Stage data have been used to address the hypothesis that the inverse relation between BMI and risk of breast cancer in young women was due to detection bias. Only one of the published reports on weight gain presented stage-related results (Brinton and Swanson, 1992). This study among women participating in a breast cancer screening programme found an inverse relation for both invasive and non-invasive cancers, but it did not stratify by stage among the invasive cases, and a large proportion of these cancers were screen-detected and early-stage. All six studies in young women that reported stage-specific information on current weight or BMI found an inverse association with risk of early-stage cancer, but little or no relation with risk of later stage cancer (Willett et al, 1985; Swanson et al, 1989; Tretli, 1989; Harris et al, 1992; Ursin et al, 1994; Taioli et al, 1995). The relative consistency in the literature for this finding suggests either that the effect of adiposity may truly be confined to early-stage cancers or that the issue has not been adequately researched. Our results are also consistent with the single study of premenopausal breast cancer that reported risks separately by grade, finding no effect of BMI on risk of poorly differentiated cancers (Willett et al, 1985). Our finding of no variation in effect by oestrogen receptor status is consistent with the study that examined this issue (Hislop and Coldman, 1986).

Except possibly for effects of overweight during adolescence on breast cancer risk in young women, evidence is insufficient for a conclusion about the effects of adolescent body size. Four other studies (Choi et al, 1978; Hislop et al, 1986; Le Marchand et al, 1988; Ursin et al, 1994) found as we did that women who were heavier in adolescence were at lower risk, although two have not (Brinton and Swanson, 1992; Franceschi et al, 1996), and one (Pryor et al, 1989) reported reduced risk. Our finding that women who recalled being much shorter were at reduced risk is consistent with one study (Brinton and Swanson, 1992), but not another (Le Marchand et al, 1988). One study reported increased risk with relative weight gain in adolescence (Ursin et al, 1994), and another reported no evidence of an effect (Brinton and Swanson, 1992). Our finding of a possible inverse relation between breast cancer risk and growth in height late in adolescence is consistent with the only other study (Li et al, 1997) that has examined this issue. Error in the recall of adolescent body size is likely to contribute to null findings and to inconsistencies among studies.

Although subject to the important limitation of accuracy of recall of earlier body size information and a few potential limitations of case control studies, our study has several strengths for an examination of this topic. The study was population-based, response rates were relatively high and most cases and controls were interviewed within a few months of their reference dates. The sample size was larger than many others, allowing for more detailed examination of dose–response relations. In addition, we were able to consider in our analysis a number of factors that might have confounded or biased the findings, including mammography history, method of cancer detection and tumour pathologic characteristics. If overweight women were less interested than lower weight women in participating as controls in this study, we may have underestimated the reduction in risk with weight gain. Among premenopausal women with breast cancer, overweight is associated with later stage at diagnosis and poorer survival (Holmberg et al, 1994; Huang et al, 1997) which may lead to a lower interview response rate among heavier, later stage cases and an overestimate of the inverse relation between weight gain and risk, particularly with late-stage cancer. However, a small proportion of our cases were non-respondents because of death or illness, and we found no relation between weight gain and risk of late-stage cancer. Cohort studies of breast cancer risk in young women have found that risk is reduced with adult weight gain (London et al, 1989) and that the effects of adiposity are confined to early-stage (Willett et al, 1985; Tretli et al, 1989) and low-grade (Willett et al, 1985) cancers.

Reasons why weight gain may result in reduced risk of breast cancer in young women are not well understood. Adult weight gain primarily reflects change in body fat (Forbes and Reina, 1970; Ballard-Barbash, 1994), and some researchers have suggested that it may be a better measure of adult adiposity than is BMI (Ziegler, 1997). In postmenopausal women, adiposity increases availability of reproductive hormones, insulin and other hormones and growth factors that may increase breast cell proliferation and cancer risk (Ballard-Barbash, 1994; Stoll et al, 1994). In premenopausal women, progesterone (Westoff et al, 1996) and oestrogen levels (Potischman et al, 1996) may be reduced in heavier women, which could explain the reduced risk. In addition, both very heavy and very light weight women have been found to be more likely to have irregular cycles (Willett et al, 1985), and we found they were less likely to have ever had regular menstrual cycles. Just as early physical maturation is associated with increased risk (Stoll et al, 1994), attainment of adult height late in the teen years may result in reduced risk (Li et al, 1997). However, for many of these potential explanations, evidence is inconsistent or limited. There are few studies of reproductive hormones in relation to body size, sample sizes are small, and evidence is

© 1999 Cancer Research Campaign inconsistent (Potischman et al, 1996). Childhood adiposity is associated with early puberty (Stoll et al, 1994), which is associated with increased risk. In our study, we did not observe a relation between recalled adolescent body size and cycle regularity. Also, although insulin levels may be positively associated with risk of breast cancer in young women (Del Giudice et al, 1998), in young women insulin levels are positively associated cross-sectionally with adiposity and longitudinally with subsequent weight gain (Folsom et al, 1998). Finally, in this study as well as others, statistical adjustment is made for several factors, including age at menarche and menstrual cycle regularity, thought to mediate or be closely associated with some of these explanatory factors.

Explanations for the inverse association between weight gain and risk of low-grade, but not high-grade, cancers are also not clear. For example, if the relation between breast cancer risk and adiposity is due to the relations between adiposity and reproductive hormones, it is uncertain why weight gain or adiposity might vary depending upon grade-related tumour biology, but not upon oestrogen receptor status.

In conclusion, our finding that adult weight gain was associated with reduced risk of early-stage, lower grade breast cancer, but not later stage, higher grade cancer suggests that weight gain may affect risk of less aggressive, but not more aggressive breast cancer in young women. This finding is consistent with most published epidemiologic studies on BMI and risk of breast cancers of different stages in young women. Our finding of a curvilinear relation between risk and adiposity in adolescence and at age 20, the inconsistencies in epidemiological studies of the effects of adolescent and early adult body size on risk, and the inconsistencies in clinical studies of relations between adult body size and hormonal characteristics suggest that these relations may be complex. Additional research in these areas will help improve our understanding of why the effects of adiposity on breast cancer risk in young women differs from the effects in older women. Future epidemiological research on breast cancer in young women may yield more consistent findings if data collection includes medical record or school record data as well as recalled information on body size, if analysis considers the possibility of complex, possibly non-linear relations, and if additional information on the biological characteristics of the breast cancers is incorporated into the studies.

### ACKNOWLEDGEMENTS

This study was supported in part by contracts from the National Cancer Institute, USA to Emory University (N01-CP 95604), the Fred Hutchinson Cancer Research Center (N01-CP-95671), and the New Jersey State Health Department of Health and Senior Services (N01-CP-95672). We gratefully acknowledge the contributions of study interviewers, managers and other staff, and thank the women who graciously agreed to participate in this study.

### REFERENCES

Ballard-Barbash R (1994) Anthropometry and breast cancer: body size – a moving target. *Cancer* **74**: 1090–1100

Breslow NE and Day NE (1980) *Statistical Methods in Cancer Research Vol. 1, The Analysis of Case Control Studies.* International Agency for Research on Cancer: Lyon

Brinton LA, Daling JR, Liff J, Schoenberg JB, Malone KE, Stanford JL, Coates RJ, Gammon MD, Hanson L and Hoover RN (1995) Oral contraceptives and breast cancer risk among younger women. *J Natl Cancer Inst* **87**: 827–835

Brinton LA and Swanson CA (1992) Height and weight at various ages and risk of breast cancer. *Ann Epidemiol* **2**: 597–609

Casey VA, Dwyer JT, Berkey CS, Coleman KA and Gardner IV (1991) Long-term memory of body weight and past weight satisfaction: a longitudinal follow-up study. *Am J Clin Nutr* **53**: 1493–1498

Choi NW, Howe GR, Miller AB, Matthews V, Morgan RW, Munan L, Burch JD, Feather J, Jain M and Kelley A (1978) An epidemiologic study of breast cancer. *Am J Epidemiol* **107**: 510–521

Chu SY, Lee NC, Wingo PA, Senie RT, Greenberg RS and Peterson HB (1991) The relationship between body mass and breast cancer among women enrolled in the Cancer and Steroid Hormone Study. *J Clin Epidemiol* **44**: 197–206

Del Giudice ME, Fantus IG, Ezzat S, McKeown-Eyssen G, Page D and Goodwin PJ (1998) Insulin and related factors in premenopausal breast cancer risk. *Breast Cancer Res Treat* **47**: 111–120

Folsom AR, Vitelli LL, Lewis CE, Schreiner PJ, Watson RL and Wagenknecht LE (1998) Is fasting insulin concentration inversely associated with rate of weight gain? Contrasting findings from the CARDIA and ARIC study cohorts. *Int J Obesity* **22**: 48–54

Forbes GM and Reina JC 1970 Adult lean body mass declines with age: some longitudinal observations. *Metabolism* **19**: 653–663

Franceschi S, Favero A, La Vecchia C, Baron AE, Negri E, Dal Maso L, Giacosa A, Montella M, Conti E and Amadori D (1996) Body size indices and breast cancer risk before and after menopause. *Int J Cancer* **65**: 181–186

Gammon MD, Schoenberg JB, Britton JA, Kelsey JL, Coates Ralph J, Brogan D, Potischman N, Swanson CA, Daling JR, Stanford JL and Brinton LA (1998) Recreational physical activity and breast cancer risk among women under age 45 years. *Am J Epidemiol* **147**: 273–280

Habel LA and Stanford JL (1993) Hormone receptors and breast cancer. *Epidemiol Rev* 1993, **15**: 209–219

Harris RE, Namboodiri KK and Wynder EL (1992) Breast cancer risk: effects of estrogen replacement therapy and body mass. *J Natl Cancer Inst* **84**: 1575–1582

Hislop TG and Coldman AJ (1986) Overweight and changes in weight throughout adult life in breast cancer etiology: a case-control study (letter). *Am J Epidemiol* **124**: 493–494

Hislop TG, Coldman AJ, Elwood JM, Brauer G and Kan L (1986) Childhood and recent eating patterns and risk of breast cancer. *Cancer Detection Prevention* **9**: 47–58

Holmberg L, Lund E, Bergstrom R, Adami HO and Meirik O (1994) Oral contraceptives and prognosis in breast cancer: effects of duration, latency, recency, age at first use and relation to parity and body mass index in young women with breast cancer. *Eur J Cancer* **30A**: 351–354

Huang Z, Hankinson SE, Colditz GA, Stampfer MJ, Hunter DJ, Manson JE, Hennekens CH, Rosner B, Speizer FE and Willett WC (1997) Dual effects of weight and weight gain on breast cancer risk. *JAMA* **278**: 1407–1411

Le Marchand L, Kolonel LN, Earle ME and Mi MP (1988) Body size at different periods of life and breast cancer risk. *Am J Epidemiol* **128**: 137–152

Li CI, Malone KE, White E and Daling JR (1997) Age when maximum height is reached as a risk factor for breast cancer among young US women. *Epidemiology* **8**: 559–565

London SJ, Colditz GA, Stampfer MJ, Willett WC, Rosner B and Speizer FE (1989) Prospective study of relative weight, height, and risk of breast cancer. *JAMA* **262**: 2853–2858

Lubin F, Ruder AM, Wax Y and Modan B (1985) Overweight and changes in weight throughout adult life in breast cancer etiology. A case-control study. *Am J Epidemiol* **122**: 579–588

Lund E, Adami HO, Bergstrom R and Meirik O (1990) Anthropometric measures and breast cancer in young women. *Cancer Causes Control* **1**: 169–172

Mannisto S, Pietinen P, Pyy M, Palmgren J, Eskelinen M and Uusitupa M (1996) Body-size indicators and risk of breast cancer according to menopause and estrogen-receptor status. *Int J Cancer* **68**: 8–13

Paffenbarger R, Kampert JB, Chang H-G (1980) Characteristics that predict risk of breast cancer before and after the menopause. *Am J Epidemiol* **112**: 259–268

Pathak DR and Whittemore AS (1992) Combined effects of body size, parity, and menstrual events in seven countries. *Am J Epidemiol* **135**: 153–168

Percy C, van Holten V and Muir C (1990) *International Classification of Diseases for Oncology* 2nd edn. World Health Organization: Geneva

Potischman N, Swanson CA, Siiteri P and Hoover RN (1996) Reversal of relation between body mass and endogenous estrogen concentrations with menopausal status. *J Natl Cancer Inst* **88**: 756–758

Potischman N, Swanson CA, Coates RJ, Weiss HA, Brogan DR, Stanford JL, Schoenberg JB, Gammon MD and Brinton LA (1997) Dietary relationships with early onset (under age 45) breast cancer in a case-control study in the

© 1999 Cancer Research Campaign

United States: influence of chemotherapy treatment. *Cancer Causes Control* **8**: 713–721
Pryor M, Slattery ML, Robison LM and Egger M (1989) Adolescent diet and breast cancer in Utah. *Cancer Res* **49**: 2161–2167
Radimer K, Siskind V, Bain C and Scholfield F (1993) Relation between anthropometric indicators and risk of breast cancer among Australian women. *Am J Epidemiol* **138**: 77–89
Ries LAG, Kosary CL, Hankey BF, Miller BA, Harras A and Edwards BK (eds) (1997) SEER Cancer Statistics Review, 1973–1994. NIH Pub. No. 97–2789. National Cancer Institute: Bethesda, MD
SAS (1992) *SAS User's Guide, version 6*, 4th edn. SAS Institute, Inc: Cary, NC
Stoll BA, Vatten LJ and Kvinnsland S (1994) Does early physical maturity influence breast cancer risk? *Act Oncol* **33**: 171–176
Swanson CA, Brinton LA, Taylor PR, Licitra LM, Ziegler RG and Schairer C (1989) Body size and breast cancer risk assessed in women participating in the Breast Cancer Detection Demonstration Project. *Am J Epidemiol* **130**: 1133–1141
Swanson CA, Coates RJ, Schoenberg JB, Malone KE, Gammon MD, Stanford JL, Shorr IJ, Potischman NA and Brinton LA (1996) Body size and breast cancer risk among women under age 45 years. *Am J Epidemiol* **143**: 698–706
Tabar L, Fagerberg G, Chen HH, Duffy SW and Gad A (1996) Tumour development, histology and grade of breast cancers: prognosis and progression. *Int J Cancer* **66**: 413–419
Taioli E, Barone J and Wynder EL (1995) A case-control study on breast cancer and body mass. *Eur J Cancer* **31A**: 723–728
Trentham-Dietz A, Newcomb PA, Storer BE, Longnecker MP, Baron J, Greenberg ER and Willett WC (1997) Body size and risk of breast cancer. *Am J Epidemiol* **145**: 1011–1019
Tretli S (1989) Height and weight in relation to breast cancer morbidity and mortality. A prospective study of 570 000 women in Norway. *Int J Cancer* **44**: 23–30
Troisi R, Weiss HA, Hoover RN, Potischman N, Swanson CA, Brogan DR, Coates RJ, Gammon MD, Malone KE, Daling JR and Brinton LA (1998) Pregnancy characteristics and maternal risk of breast cancer. *Epidemiology* **9**: 641–648
Ursin G, Paganini-Hill A, Siemiatycki J, Thompson WD and Haile RW (1994) Early adult body weight, body mass index, and premenopausal bilateral breast cancer: data from a case-control study. *Breast Cancer Res Treat* **33**: 75–82
Ursin G, Longnecker MP, Haile RW and Greenland S (1995) A meta-analysis of body mass index and risk of premenopausal breast cancer. *Epidemiology* **6**: 137–141
Westoff C, Gentile G, Lee J, Zacur H and Helbig D (1996) Predictors of ovarian steroid secretion in reproductive-age women. *Am J Epidemiol* **144**: 381–388
Willett WC, Browne ML, Bain C, Lipnick RJ, Stampfer MJ, Rosner B, Colditz GFA, Hennekens CH and Speizer FE (1985) Relative weight and risk of breast cancer among premenopausal women. *Am J Epidemiol* **122**: 731–740
Ziegler RG (1997) Anthropometry and breast cancer. *J Nutr* 127s: 924s–928s
Ziegler RG, Hoover RN, Nomura AM, West DW, Wu AH, Pike MC, Lake AJ, Horn-Ross PL, Kolonel LN, Siiteri PK and Fraumeni JF (1996) Relative weight, weight change, height, and breast cancer risk in Asian-American women. *J Natl Cancer Inst* **88**: 65–70

 *© 1999 Cancer Research Campaign*
